# Supplementary material for: Patterns of Intron Gain and Loss in Fungi
Source: PLoS Biol. 2004 Nov 30;2(12):e422. doi: 10.1371/journal.pbio.0020422 (PMC532390; doi:10.1371/journal.pbio.0020422)
Supplement: Table S1 — Also available at http://genes.mit.edu/NielsenEtAl/. (4.3 MB ZIP). [file pbio.0020422.st001.zip › NielsenEtAl/html/626.html]

AN9411.1.NCU01765.1.MG03155.1.FG05198.1


```
 CLUSTAL W (1.82) Multiple Sequence Alignments - Introns Inserted


Sequence 1: NCU01765.1	744 aa
Sequence 2: MG03155.1	729 aa
Sequence 3: FG05198.1	760 aa
Sequence 4: AN9411.1	738 aa
Alignment Length: 762 aa
Number Identitical Residues: 484 aa
Alignment Score (without introns) 22562


MG03155.1 	MLRQTLARSAWRTGRHAAK--ASRAFSATTQRPAEVELTIA1G-------~-SALIQACE
NCU01765.1	MLRSTLSRSAWRTGRHQAARNASRAFSATAQRPAEVELTID1GKKVSIEA1GSALIQACE
FG05198.1 	MLRNTLARSAWRTSRRAVN--VSRTFATTSQRPAEVELTID1GKKVSIEA1GSALIQACE
AN9411.1  	-MRPQLFRAAARSVRVPKVN-TARTFVTTVPRSAEVELTID1GKKVSVEA1GSALIQACE
          	 :*  * *:* *: *      .:*:* :*  *.*******  *.. : .: .********

MG03155.1 	KAGATVPR2-----~KLMIAGNC~RMCLVEVEKVPKPVASCAWPVQPGMVVKTNSPIAHK
NCU01765.1	KAGVTIPR2YCYHE2KLMIAGNC~RMCLVEVEKVPKPVASCAWPVQPGMVVKTNSPLTHK
FG05198.1 	KAGSTVPR2YCYHE2KLMIAGNC~RMCLVEVERAPKPVASCAWPVQPGMVVKTNSPLTHK
AN9411.1  	KAGATIPR2YCYHE2KLMIAGNC1RMCLVEVERAPKPVASCAWPVQPGMNVKTDSPLVHK
          	*** *:**  .  . ******** ********:.*************** ***:**:.**

MG03155.1 	AREGVMEFLLANHPLDCPVCDQGGECDLQDQSMRYGADRGRFHEIGGKRAVEDKNIGPLI
NCU01765.1	AREGVMEFLLANHPLDCPICDQGGECDLQDQSMRYGGDRGRFHEVGGKRAVEDKNMGPLI
FG05198.1 	AREGVMEFLLANHPLDCPVCDQGGECDLQDQSMRYGADRGRFHEVGGKRAVEDKNIGPLI
AN9411.1  	AREGVMEFLLANHPLDCPICDQGGECDLQDQSMRYGADRGRFHELGGKRAIEDKNIGPLV
          	******************:*****************.*******:*****:****:***:

MG03155.1 	KTSMNRCIHCTRCVRFANDIAGANEMGSTGRGNDLQIGTYLEKNLDSELSGNVIDLCPVG
NCU01765.1	KTSMNRCIQCTRCVRFANDIAGAPELGSTGRGNDLQIGTYLEKNLDSELSGNVIDLCPVG
FG05198.1 	KTSMNRCIHCTRCIRFSNDIAGAPEMGSTGRGNDIQIGTYLEQNLDTELSGNVIDLCPVG
AN9411.1  	KTSMNRCIQCTRCVRFMNDVAGAPELGTTGRGNDMQIGTYLEQNLDTELSGNIIDLCPVG
          	********:****:** **:*** *:*:******:*******:***:*****:*******

MG03155.1 	ALTSKPYAFRARPWELKKTESIDVMDALGANIRVDSRGLEVMRILPRLNDDVNEEWIDDK
NCU01765.1	ALTSKPYAFRARPWELKKTESIDVLDGLGSNIRVDTRGLEVMRILPRLNDEVNEEWINDK
FG05198.1 	ALTSKPYAFRARPWELKHTESIDVLNGLGSNIRVDSRGLEVMRILPRLNDDVNEEWIDDK
AN9411.1  	ALTSKPYAFRARPWELKHTESIDVHDALGSNVRIDTRGMEVMRVLPRLNDDINEEWINDK
          	*****************:****** :.**:*:*:*:**:****:******::*****:**

MG03155.1 	TRFACDGLRTQRLTLPLVRKEGKFEPASWEQALNEVAENFARIAPKGNEFKVIAGQLTEV
NCU01765.1	TRFACDGLKTQRLTIPLVRREGKFEPASWDQALTEIAHAYQTLNPQGNEFKAIAGQLTEV
FG05198.1 	TRFACDGLKTQRLTMPLVRREGRFEPSDWEEALTEIGRAYQIKNPQGNEFKIIAGALTEV
AN9411.1  	SRFACDGLKTQRLTTPLIRQEGKFVPATWEQALTEIASAYQKLQLKENEFKAISGHLVDA
          	:*******:***** **:*:**:* *: *::**.*:.  :     : **** *:* *.:.

MG03155.1 	ESLVAMKDMANKLGSENLALDMPSGSQPIAHGVDVRSNYLFNSRIAGVEEADAILIVGSN
NCU01765.1	ESLVAMKDLANRLGSENLALDMPSGHKPLAHGVDVRSNYIFNSSIVGIESADVILLVGTN
FG05198.1 	ESLVVAKDMANKLGSDNLALDTPTGSQPLAHGVDVRSNFLFNSQIWGIEEADAILIVGSN
AN9411.1  	ETMVAMKDLANKLGSDNLALDQPGGSSPIAHGVDIRSNYLFNSKIYGIEEADAILLVATN
          	*::*. **:**:***:***** * * .*:*****:***::*** * *:*.**.**:*.:*

MG03155.1 	PRHEAAVLNARIRKQWLRSDLEIGVVGETWDSTFEFEHLGTDAAALKKALAGPFGKKLQS
NCU01765.1	PRHEAAVLNARIRKQWLRSDLEIGVVGQTWDSTFEFEHLGTDHAALQKALEGDFGKKLQS
FG05198.1 	PRHEAAVLNARIRKQWLRSDLEIGLVGETFDSTFEFEHFGADHAALKTALSGPFGETLKN
AN9411.1  	PRHEASVLNARIRKQYLRSDLEIGLVGEAFESTFDFEHLGSDVAALKAALSGNFGTKLAS
          	*****:*********:********:**::::***:***:*:* ***: ** * ** .* .

MG03155.1 	AKRPMIILGSGVTDHADAKAFYEMVGAFVDKNAANFMTPEWNGYNVLQRSASRAGAFEVG
NCU01765.1	AKNPMIIVGSGVTDHGDANAFYETVGKFVDSNASNFLTEEWNGYNVLQRAASRVGAFEVG
FG05198.1 	AKRPMIIVGSGVTDHADAKAYYETIGTFVDKHASNFRTEEWQGYNVLQREASRAGAFEVG
AN9411.1  	AKRPMIIVGSAAAEHKDSKAIFEAVGSFVEKHASNFNTPEWQGYNVLQRAASRAAAYEIG
          	**.****:**..::* *::* :* :* **:.:*:** * **:******* ***..*:*:*

MG03155.1 	FVTPSAAVAETKPKIVWLLGADDFQAADIPKDAFVIYQGHHGDRGAEIADVVLPGAAYTE
NCU01765.1	FTVPSAEIAQTKPKFVWLLGADEFNEADIPKDAFIVYQGHHGDRGAQIADIVLPGAAYTE
FG05198.1 	FTTPSAEVAQTKPKFVWLLGADDVKEADIPKDAFVVYQGHHGDKGAQIADIVLPGAAYTE
AN9411.1  	FTTPSPEVAQTTPKMVWLLGADEINQSEIPREAFVIYQGHHGDRGAQIADVVLPGAAYTE
          	*..**. :*:*.**:*******:.: ::**::**::*******:**:***:*********

MG03155.1 	KSATYINTEGRVQMTRAATSLPGAARTDWKIIRAASEFLGVPLPYDDLAMVRDRMVEISP
NCU01765.1	KAGTYVNTEGRVQMTRAATGLPGAARTDWKILRAVSEYLGVRLPYDDVAQLRDRMVEISP
FG05198.1 	KAGTYINTEGRVQMTRAATSLPGASRTDWKILRAASEFLGAPLPYDDVAMVRDRMVEISP
AN9411.1  	KSGTYINTEGRVQVTRAATSMPGASRDDWKIIRAASEFLNTPLPYDDIEALRDRMEEISP
          	*:.**:*******:*****.:***:* ****:**.**:*.. *****:  :**** ****

MG03155.1 	ALAAYDVVEPVALQQLSKVQLVDQNKGSKPTNAALKHAVENFYFTDVISR----------
NCU01765.1	ALSSYDIIEPPSLQQLSKVQLVEQNQGATATNEPLKKVIENFYFTDAISR----------
FG05198.1 	ALAAYDVVEPVALPALSKVQLVDQNKGAKVTGAPLKKVIDNFYFTDVISRRYVNLIYHVP
AN9411.1  	VMRRYDVVEPTSLAQLSKVQLVDQNKGSQVTNAPFKKVIEDFYFTDSISR----------
          	.:  **::** :*  *******:**:*:  *. .:*:.:::***** ***          

MG03155.1 	--------S2SPTMARCSAAKETGNPKTDFMAPGMTEDKPMGQVAYGS
NCU01765.1	--------S2SPTMARCSAAKKTGDPRTNFMAPGMEEDRPMGQIAYGA
FG05198.1 	EFPTNNLFS~SPTMARCSAAKATGDARTNFMAPGMEEDKPMGQVEYGV
AN9411.1  	--------S2SPTMARASAAKVSGNPDVNYMSTGGEVSP---QHMYG-
          	        * ******.**** :*:. .::*:.*   .    *  **
```
